# Additional years of Australian Rural Clinical School undergraduate training is associated with rural practice

**DOI:** 10.1186/1472-6920-13-37

**Published:** 2013-03-07

**Authors:** Lesley Forster, Hassan Assareh, Lisa D Watts, Craig S McLachlan

**Affiliations:** 1 Rural Clinical School, Faculty of Medicine, University of New South Wales, Sydney, NSW 2052, Australia; 2 Simpson Centre for Health Services Research, Australian Institute of Health Innovation, Faculty of Medicine, University of New South Wales, Sydney, NSW 2052, Australia

**Keywords:** Undergraduate medical education, Rural medical education, Rural clinical school, Rural exposure, Medical workforce, Logistic regression models

## Abstract

**Background:**

To understand the influence of the number of years spent at an Australian rural clinical school (RCS) on graduate *current*, *preferred current* and *intended* location for rural workforce practice.

**Methods:**

Retrospective online survey of medical graduates who spent 1–3 years of their undergraduate training in the University of New South Wales (UNSW) Rural Clinical School. Associations with factors (gender, rural versus non-rural entry, conscription versus non-conscript and number of years of RCS attendance) influencing *current*, *preferred current* and *intended* locations were assessed using χ2 test. Factors that were considered significant at P < 0.1 were entered into a logistic regression model for further analysis.

**Results:**

214 graduates responded to the online survey. Graduates with three years of previous RCS training were more likely to indicate rural areas as their *preferred current* work location, than their colleagues who spent one year at an RCS campus (OR = 3.0, 95% CI = 1.2-7.4, P = 0.015). Also RCS graduates that spent three years at an RCS were more likely to *intend* to take up rural medical practice after completion of training compared to the graduates with one year of rural placement (OR = 5.1, 95% CI = 1.8-14.2, P = 0.002). Non-rural medicine entry graduates who spent three years at rural campuses were more likely to take up rural practice compared to those who spent just one year at a rural campus (OR = 8.4, 95% CI = 2.1-33.5, P = 0.002).

**Conclusions:**

Increasing the length of time beyond a year at an Australian RCS campus for undergraduate medical students is associated with *current* work location, *preferred current* work location and *intended* work location in a rural area. Spending three years in a RCS significantly increases the likelihood of rural career *intentions* of non-rural students.

## Background

Attracting doctors to serve in isolated areas is a problem that has been identified in many developed and developing nations. To address doctor shortages in rural and remote Australia the Australian government established the Rural Clinical School (RCS) Program in 2001 to provide medical students with exposure to rural medicine and rural lifestyle during the clinical years of their training [1]. This is one program amongst a suite of measures, including the recruitment of medical students with a rural background, aimed at increasing the rural medical workforce.

The Australian Department of Health and Ageing has invested heavily in the RCS program and there are currently 17 Rural Clinical Schools across the country [1]. A funding requirement is that a minimum of 25% of domestic students spend at least one year of their clinical training in a rural area in the belief that extended rural exposure will increase students’ interest in rural medical practice [1]. One of the challenges in evaluating national outcomes of the RCS program is that the nature and duration of the students’ rural exposure can vary according to the particular clinical training model adopted by each Australian medical school. Evidence of the specific effect of ‘rural exposure’ has been somewhat inconclusive [2].

The study discussed in this paper aims to contribute to our understanding of the effect of rural exposure by investigating one of the distinctive features of the University of New South Wales (UNSW) RCS program, which is that students can undertake up to three clinical years (well above the government requirement) of their six year undergraduate program in RCS campuses in the state of New South Wales. In this longitudinal study, known as the “UNSW Rural Clinical School Graduate Destinations Study” we examine the actual career destinations of our graduates and importantly whether there is an association between the extended amount of time spent in the RCS, and rural practice.

Our aim is to determine whether increasing the length of time of RCS experience from 1 year to 2 years or 1 to 3 years yields an increasing positive impact on rural work choice (*current*, *preferred current* and *intended*) in both conscripted versus non-conscripted, and rural versus non-rural entry medical students.

## Methods

**Figure 1:**
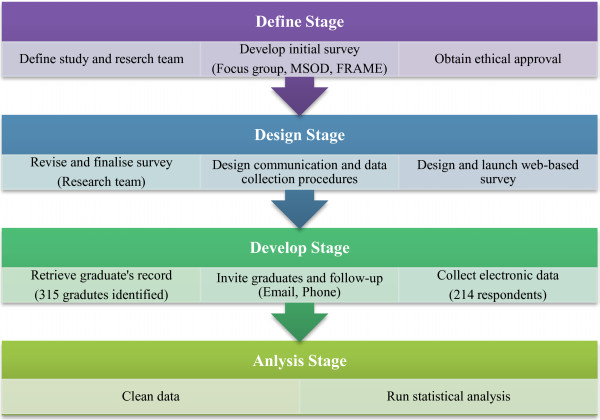
Flowchart of study stages.

This study was approved by the Human Research Ethics Committee of UNSW. Three hundred and fifteen graduates were contacted to complete an online questionnaire. The online Graduate Destinations Survey (GDS) questionnaire was designed by utilizing UNSW RCS staff as part of a focus group to derive relevant questions. Additionally, questions were designed to be not too dissimilar to other published Australian rural medical school questionnaires [3] (Figure 1). The key questions that were modelled in this study are summarized in Table 1. The key difference between our GDS and other published studies is to examine work locations of rural clinical school graduates with various combinations of 1–3 years of undergraduate training.

**Table 1:** Key GDS survey questions used in the model (out of a total of 21 questions)

**Cohort**	**In what year did you complete your medical degree?**
**Rural/non-rural background**	Through which entry scheme did you enter the medicine program?
**Number of years in Rural Clinical School**	Which clinical school campus were you assigned to in year 3, 4, 5 & 6 (rural or metropolitan)?
**Conscription status**	Did you choose to study at the Rural Clinical School?
**Position**	What is your current primary position? (intern, resident, registrar, GP trainee, GP, Specialist)
**Current work location**	How would you categorise the region in which you primarily work at present (inner urban, outer metropolitan, regional, rural, remote)?
**Preferred work location**	What location would you like to be working now (inner urban, outer metropolitan, regional, rural, remote)?
**Intended work location**	What location do you intend to work in after completing all your training (and bonding period if applicable) (inner urban, outer metropolitan, regional, rural, remote)?

As the RCS program has been running just over a decade and due to the relative youth of our graduates, we asked graduates to self-report their *current* practice location (where they currently work); their *preferred current* practice location (in recognition that junior doctors may prefer a rural location were it not for the constraints of some training schemes and job opportunities); and their *intended* practice location (after completion of training). These locations were based on five regional categories that included Inner urban, Outer metropolitan, Regional, Rural and Remote areas. With respect to statistical analysis, regions were collapsed and re-categorized into two main demographic areas 1) Urban, consisting of the first two regions (included Inner urban, Outer metropolitan); and 2) Rural, the last three categories (Regional, Rural and Remote areas).

Gender, student entry scheme (rural and non-rural), conscript attendance at RCS, and the length of stay (one, two or three years) were considered in the association analysis. These factors were modelled as potential contributors for choosing *current*, *preferred current* and *intended* career rural locations.

Rural background is known to be a predictor of intention to practice in a rural area [4,5], and for the purpose of this study it was determined by whether graduates had entered the UNSW Medicine program under the Rural Entry Scheme. To be eligible for the Rural Entry Scheme, students must have lived in a rural area in Australia for a minimum of five years, the rest of the cohort was defined as non-rural entry. Conscription to attend an RCS was considered to be an important potential influence on graduates’ motivation to practice in a rural area [6]. For the purpose of the present study we have defined conscription as medical students who did not volunteer to attend an RCS but were allocated for 1 year. Beyond 1 year there was no conscription for any medical student attending an RCS.

Data were analysed using SPSS version 20.0 for Windows. Associations of the proposed factors with the current, preferred and intended locations were assessed using χ2 test. Factors that were considered significant at P < 0.1 were entered into a logistic regression model to obtain adjusted odds ratio for further analysis. Missing data was assumed to be missing at random and therefore data available was used for analysis, actual student numbers used in all data summary tables are provided, P <0.05 was considered statistically significant.

## Results

**Figure 2:**
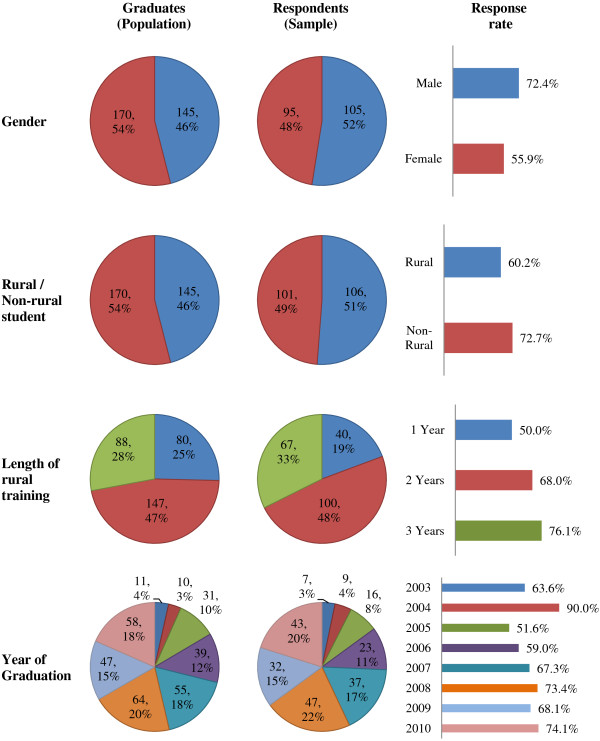
**Distribution of a) RCS graduates, b) respondents to Graduate Destination Survey, and c) response rates of cohorts between 2003–2010.** Individual colours represent graduating years (bottom panel).

Of 315 identified graduates between the years 2003–2010, we observed a response rate of 68%, where 214 graduates responded to the online survey. Within each demographic characteristic in the model, the observed response rate was 50-90% and therefore each subgroup had no inherent bias and was well balanced (Figure 2).

**Figure 3:**
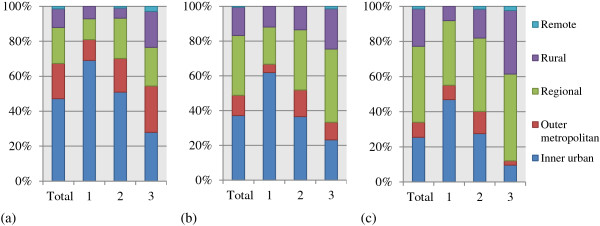
**Percentage of graduates and who spent one, two and three years at the rural campuses and a) current workplace, b) preferred current workplace, and c) intended workplace.** (Total bars depict combined proportions of 1–3 years).

As shown in Figure 3, the combined proportion of 1–3 years show that 26% of respondents are currently working in rural areas. However more than 50% of respondents expressed their preference to be currently working in a rural area; and moreover 67% of respondents intended to take up rural practice after completing all medical training. Of the respondents 85% indicated they had chosen to attend the RCS (non-conscripts) for their clinical placement.

Graduates’ rural work locations (*current*, *preferred current and intended*), increased proportionally to the number of years at rural campuses, as seen in Figure 3.

Additionally, there was a significant association between the entry scheme (rural and non-rural entry) and rural practice which were modelled on the basis of *current*, *preferred current* or *intended* work locations; see Table 2. Conscript allocation to RCS was also found to be significantly associated with *preferred current* and *intended* work locations (Table 2). Gender was found to be a non-significant factor in choosing *current*, *preferred current* and also *intended* future locations for medical practice (Table 2).

**Table 2:** Frequency and association analysis of the proposed factors with practice locations (current, preferred, and intended) of medical graduates

**Factors**	**Frequency (%) of graduates**		**P**-**value (χ2 test)**
Current location			
	Urban	Rural	
Gender (n = 200)			0.570
Male	81 (40%)	24 (12%)	
Female	70 (35%)	25 (13%)	
RCS attendance (n = 207)			0.969
Conscript	23 (11%)	8 (4%)	
Non-Conscript	130 (63%)	46 (22%)	
Entry Scheme (n = 207)			0.001
Non-Rural	85 (41%)	16 (8%)	
Rural	68 (33%)	38 (18%)	
Length of placement (n = 207)			0.024
1 year	34 (16%)	6 (3%)	
2 years	77 (38%)	23 (11%)	
3 years	42 (20%)	25 (12%)	
Preferred current location			
	Urban	Rural	
Gender (n = 206)			0.586
Male	57 (28%)	51 (25%)	
Female	48 (23%)	50 (24%)	
RCS attendance (n = 213)			0.004
Conscript	23 (11%)	8 (4%)	
Non-Conscript	84 (40%)	98 (46%)	
Entry Scheme (n = 213)			<0.001
Non-Rural	68 (32%)	35 (17%)	
Rural	39 (18%)	71 (33%)	
Length of placement (n = 213)			0.001
1 year	29 (14%)	12 (5%)	
2 years	55 (26%)	48 (23%)	
3 years	23 (11%)	46 (21%)	
Intended work location after completion of training			
	Urban	Rural	
Gender (n = 205)			0.372
Male	33 (16%)	74 (35%)	
Female	36 (17%)	62 (32%)	
RCS attendance			<0.001
Conscript	19 (9%)	11 (5%)	
Non-Conscript	52 (25%)	130 (61%)	
Entry Scheme (n = 212)			<0.001
Non-Rural	53 (25%)	50 (24%)	
Rural	18 (8%)	91 (43%)	
Length of placement (n = 212)			<0.001
1 year	22 (11%)	19 (9%)	
2 years	41 (19%)	62 (29%)	
3 years	8 (4%)	60 (28%)	

*Rural versus Non*-*rural entry*: For graduates that entered medicine through the Rural Entry Scheme who undertook an RCS training place, the odds ratio of currently working in a rural location was 2.5 (95% CI = 1.3-5.0, P = 0.009) when compared to non-rural entry counterparts (Table 3). Adjusted odds ratios of *preferred current* and *intended* rural work locations were observed for rural graduates (OR = 2.9, 95% CI = 1.6-5.2, P < 0.001 and OR = 4.4, 95% CI = 2.2-8.7, P < 0.001, respectively), compared to non-rural counterparts.

**Table 3:** Odds ratio of different entry scheme and length of rural placement in taking up rural medical practice

**Factors**	**Odds ratio**	**Confidence interval (95%)**	**P**-**value (Wald test)**
Current work location-Rural area			
Entry Scheme			
Rural compared to non-rural	2.5	1.3-5.0	0.009
Length of rural placement			
3 years compared to 1 year	2.5	0.9-7.0	0.085
2 years compared to 1 year	1.6	0.8-3.3	0.166
Preferred current work location-Rural area			
Entry Scheme			
Rural compared to non-rural	2.9	1.6-5.2	<0.001
RCS attendance			
Non-Conscript to conscript	2.5	1.0-6.3	0.015
Length of rural placement			
3 years compared to 1 year	3.0	1.2-7.4	0.015
2 years compared to 1 year	1.7	0.7-3.8	0.191
Intended work location after completion of training- Rural area			
Entry Scheme			
Rural compared to non-rural	4.4	2.2-8.7	<0.001
RCS attendance			
Non-Conscript to conscript	3.2	1.3-7.6	0.01
Length of rural placement			
3 years compared to 1 year	5.1	1.8-14.2	0.002
2 years compared to 1 year	1.3	0.6-2.9	0.494

*Conscript versus non*-*conscript*: Graduates who attended the RCS voluntarily (non-conscript), were significantly more likely to be interested in rural practice as their *preferred current* and *intended* work locations (preferred OR = 2.5, 95% CI = 1.0-6.3, P = 0.015 and intended OR = 3.2, 95% CI = 1.3-7.6, P = 0.01, respectively).

*Length of stay* (*RCS training 1*–*3 years*): The odds ratio was 3.0 for graduates with three years of training and indicating rural practice as their *preferred current* work location compared to their colleagues who spent one year at an RCS campus (95% CI = 1.2-7.4, P = 0.015). RCS graduates who spent three years at an RCS were more likely (OR = 5.1, 95% CI = 1.8-14.2, P = 0.002) to *intend* to take up rural practice after completion of training, compared to the graduates with one year of rural placement (Table 3). The odds ratio was non-significant for two years RCS training compared to one year for all rural work locations (Table 3).

Next, we separated the graduates into two main groups of rural and non-rural and studied the contribution of number of years on all locations for each group. As a consequence, entry scheme was eliminated from the association analysis and the logistic regression models. We replicated the analysis for rural and non-rural graduates independently and the results are re-examined in this context below.

*Non*-*rural entry and length of stay*: As summarised in Table 4, the association analysis shows that *current* and *intended* work locations were significantly associated with length of stay for non-rural graduates. Non-rural students who spent three years at the RCS were more likely to *currently* work (OR = 6.9, 95% CI = 1.2-39, P = 0.028) or *intend* to work (OR = 8.4, 95% CI = 2.1-33.5, P = 0.002) in rural practice, compared to those who stayed one year. The effect of two years compared to one year, on rural practice for non-rural graduates was not significant. The effect of length of stay on *preferred current* location was not significant for non-rural graduates (χ2 P = 0.115), however the odds ratio of non-rural graduates with three years training compared to one year approached statistical significance (Wald test P = 0.083).

**Table 4:** Odds ratio of different length of rural placement in taking rural medical practice by rural and non-rural students

**Cohort**	**Factor**		**Odds ratio**	**Confidence interval (95%)**	**P**-**value (Wald test)**
Non-rural student			Current work location-Rural area		
	Length of rural placement	3 years compared to 1 year	6.9	1.2-39	0.028
	(χ2 p-value = 0.025*)	2 years compared to 1 year	1.9	0.4-10.1	0.444
			Preferred current work location-Rural area		
	RCS attendance	Non-Conscript to conscript	3.1	0.8-11.9	0.095
	(χ2 p-value = 0.052*)				
	Length of rural placement	3 years compared to 1 year	3.0	0.9-10.2	0.083
	(χ2 p-value = 0.115)	2 years compared to 1 year	1.2	0.4-3.5	0.713
			Intended work location after completion of training- Rural area		
	RCS attendance	Non-Conscript to conscript	3.2	1.0-10.5	0.058
	(χ2 p-value = 0.028*)				
	Length of rural placement	3 years compared to 1 year	8.4	2.1-33.5	0.002
	(χ2 p-value = 0.002)	2 years compared to 1 year	1.5	0.6-4.0	0.424
Rural student			Current work location-Rural area		
	Length of rural placement	3 years compared to 1 year	1.5	0.4-5.9	0.541
	(χ2 p-value = 0.883*)	2 years compared to 1 year	1.2	0.3-4.7	0.761
			Preferred current work location-Rural area		
	RCS attendance	Non-Conscript to conscript	2.3	0.6-8.3	0.206
	(χ2 p-value = 0.078*)				
	Length of rural placement	3 years compared to 1 year	3.7	1.0-13.8	0.051
	(χ2 p-value = 0.064*)	2 years compared to 1 year	2.7	0.7-9.6	0.130
			Intended work location after completion of training- Rural area		
	RCS attendance	Non-Conscript to conscript	3.9	1.0-15.8	0.055
	(χ2 p-value = 0.017*)				
	Length of rural placement	3 years compared to 1 year	2.1	0.4-12.5	0.388
	(χ2 p-value = 0.153*)	2 years compared to 1 year	0.9	0.2-4.2	0.922

* Fisher’s Exact test was reported since some of cells had expected counts less than five.

*Rural entry and length of stay*: In Table 4, the association analysis showed that *current* and *intended* rural work locations of rural graduates was not significantly influenced by the length of stay at an RCS. However, there was an effect for the number of years on *preferred current* location for rural graduates. Rural graduates who spent three years at RCS were more likely (OR = 3.7, 95% CI = 1.0-13.8, P = 0.051) to *prefer* to work at a rural location when compared to those who stayed one year.

*Non*-*rural and conscription*: Odds ratios approached statistical significance in the model for whether RCS non-conscripted non-rural graduates were more likely to choose a rural area for their *preferred current* and *intended* work area, compared to RCS conscript non-rural graduates (OR = 3.1, 95% CI = 0.8-11.9, P = 0.095 and OR = 3.2, 95% CI = 1.0-10.5, P = 0.058, respectively).

*Rural and conscription*: As above, a similar tendency was also seen for rural graduates in taking up rural practice after completing their medical training (OR = 3.2, 95% CI = 1.0-10.5, P = 0.058).

## Discussion

Our first order analysis demonstrated that increasing the number of years to 3 years in an RCS, was associated with improved outcomes for *current*, *preferred current* and *intended* rural practice locations compared to one year. These rural practice associations remained significant after adjustment for conscription/non-conscription and student entry scheme (rural/ non-rural). To date there have been a limited number of Australian studies that have addressed length of time at an RCS on workforce outcomes [7-9]. To the best of our knowledge previous Australian studies have not modelled time within an RCS beyond 2 years, on rural practice. It has been shown elsewhere that rural exposure can particularly benefit students who have an urban background [10,11] and indeed our study shows that the incremental effect of three years of rural exposure on non-rural entry students, during their final clinical years, has a greater effect on their rural practice intentions than for rural entry students, when compared to 1 year. This is an important finding showing that three years in a rural medical environment for urban students can have significant outcomes on the rural workforce, in particular with respect to *current* and *intended* work locations.

A comparable finding has been made by the Australian National University (ANU) Medical School’s 4 year medical school program [12]. ANU explored length of rural training for elective and compulsory program components on student intentions for both urban and non-urban entry on practice in a rural and remote location post-graduation. The ANU cohort had 40 students and were modelled to length of rural stay for either 6 weeks or a 1 year with an optional additional 1 year rural elective. This study found that students from non-rural backgrounds have a greater positive change in their intention to practice rurally as an effect of longer term rural placements when compared with students from rural backgrounds. Our findings also suggest that increasing the length of time in a rural community increases affiliation for future rural practice for non-rural entry students. Interestingly another study found that for non-rural entry students up to two years of rural exposure during pre-clinical training also has a beneficial outcome on rural practice. The Indiana University School of Medicine (IUSM) revealed in a recent study, that when students were exposed to the training environments of the regional campuses for 2 years of pre-clinical studies, this was a significant predictor of both medical specialty choice and practice location choice in logistic regression models that incorporated several covariates known to influence these career decisions [13].

**Figure 4:**
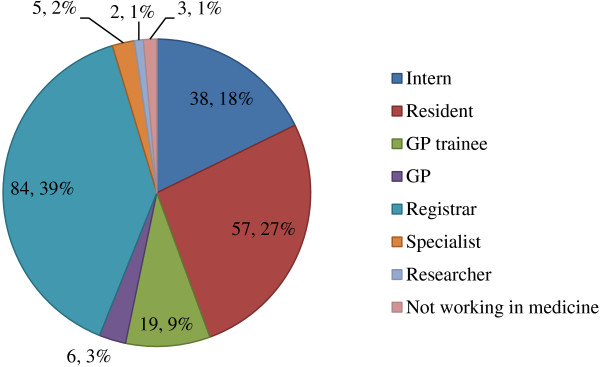
Distribution of current position of respondents.

Rabinowitz et al. [14] have suggested that medical students’ rural background and rural career plans known at the time of entrance to medical school are strongly related to practicing in a rural area three decades later. This implies that rural career intentions are relatively stable over time and can reliably predict career location and type of practice. In our study 43% of UNSW RCS rural entry graduates intend to work in rural areas, although we do not know if this was their original intention. Further longitudinal follow-up of our cohort may reveal a significant increase in the rural practice locations of RCS graduates over time. We appreciate that limited prevocational training positions available to RCS graduates and the constraints of urban-centric specialty training programs have impacted on current workforce location in our study (for distribution of the current position of respondents see Figure 4).

In our study we did not differentiate between rural background students on the basis of the size of their home town. This design was based on a previous Australian study showing no differences in rural practice location for doctors who grew up in different sized communities from very small rural through to large rural areas [15]. In our model we included the contribution of conscription and non-conscription on RCS workforce practice. Conscripts in our analysis included less than 15% of the respondents, hence limiting the potential to comment on this sub-group analysis at length, in particular when subdividing the analysis on the basis of rural and non-rural entry. Notwithstanding this limitation, we showed that rural *preferred current* and *intended locations* of non-conscript graduates of the RCS were stronger than their conscript peers. In summary our study centres around non-conscripted students beyond one year and this is associated with increased return to rural workforce. Epidemiological evidence remains lacking to address the question whether conscription reduces Australian rural workforce uptake with additional years at a rural clinical school. With our data presented in this study we cannot model outcomes beyond 1 year for conscripted students.

Students’ motivation to attend an Australian RCS is complex and involves many factors, including personal, psychological and academic influences. Factors that have been cited in an Australian context and influence attending an RCS include perceived ability to obtain better grades, better patient access, academic mentorship, personality type, peers, community, lifestyle and family. As we have alluded to, our study was not designed to provide a complex model of all the predictive factors that may influence choice to attend a rural clinical school and ultimately workforce outcomes. Our study is a starting point to explore associations with length of time in rural training on rural workforce outcomes and guide policy and decision tree modelling concerning the optimum number of years for rural clinical school training.

## Conclusion

Qualitative studies have shown that by spending an extended length of time in a rural setting students are able to immerse themselves in their local community, have a more authentic and well rounded experience of rural medicine, and envisage living and working as a rural practitioner [11,16,17]. While there may be no definitive answer to ‘how much’ rural exposure is required, the results of this study suggest that three years are associated with rural workforce outcomes for students and in particular non-rural entry students.

## Competing interests

The authors declare that they have no competing interests.

## Authors’ contributions

LF conceived of the study and provided conceptual input to the survey and final manuscript. HA provided statistical modelling of the data and wrote the results and methods section of the manuscript. LW contributed to the design of the survey and acquisition of data and contributed to the drafting of the manuscript. CM contributed to the design of the study, analysis of results and co-ordination, drafting of the manuscript and handling the review process. All authors read and approved the final manuscript.

## Pre-publication history

The pre-publication history for this paper can be accessed here:

http://www.biomedcentral.com/1472-6920/13/37/prepub
